# Heat stress and air pollution in female reproductive health: from pathological alterations to molecular mechanisms

**DOI:** 10.1080/07853890.2026.2757496

**Published:** 2026-11-05

**Authors:** Xiangyu Liu, Jiao Yu, Tianjiao Zheng, Shibo Li, Yingbo Liu, Yuhui Sun

**Affiliations:** ^a^ Department of Gynecology, the First Affiliated Hospital of Harbin Medical University, Harbin, China; ^b^ Department of Gynecology, The Second Hospital of Changsha (West Branch of Changsha Hospital for Maternal & Child Health Care), Changsha, China

**Keywords:** Environment, heat stress, air pollution, Reproduction, pathology, mechanisms

## Abstract

**Background:**

Female reproductive health is increasingly threatened by environmental degradation, particularly climate change and air pollution. This narrative review synthesizes epidemiological, pathophysiological, and mechanistic evidence on how these exposures affect female reproductive health.

**Discussion:**

Epidemiological studies associate these exposures with diminished ovarian reserve, reduced implantation and live birth rates, increased pregnancy loss, and elevated risks of preterm birth and hypertensive disorders. Pathological evidence supports impaired follicular development, defective placental vascular remodeling, and reduced endometrial receptivity. Mechanistic studies implicate oxidative stress and inflammatory activation as initiating events, with epigenetic reprogramming and dysregulated apoptosis and autophagy as downstream mediators of persistent damage. However, current evidence is limited by heterogeneous exposure assessment methods, discrepancies between animal models and human pathology, insufficient characterization of susceptible windows, and predominant focus on female factors while neglecting male contributions. Despite these limitations, the convergence of epidemiological, pathological, and molecular evidence across diverse populations and experimental systems strengthens the biological plausibility of causal links.

**Conclusion(s):**

High temperature and air pollution adversely affect female reproductive health through interconnected epidemiological, pathological, and molecular pathways. Epidemiological evidence consistently links these exposures to clinical reproductive impairments, supported by pathological findings of disrupted follicular development, placental vascular remodeling, and endometrial receptivity. Mechanistic studies implicate oxidative stress, epigenetic dysregulation, and disrupted apoptosis and autophagy as potential mediators of these effects. Future research should prioritize standardized exposure assessment, long-term prospective cohorts to identify vulnerable populations and critical windows, and translational validation of preclinical findings to inform public health policies and clinical strategies.

## KEY MESSAGES

This review presents an integrated evidence chain connecting epidemiology, pathology, and molecular mechanisms to elucidate the distinct yet interconnected effects of ambient temperature and air pollution on key female reproductive events, including ovarian, uterine, embryonic, and placental function. The article further focuses on three core molecular mechanisms, namely oxidative stress, epigenetic regulation, and dysregulated programmed cell death, in mediating these environmental impacts.

## 1. Introduction

Globally, women’s reproductive health is confronting increasingly severe challenges. According to data from multiple countries worldwide, infertility affects millions of couples, while the incidence of adverse pregnancy outcomes such as miscarriage and preterm birth remains persistently high [1,2]. Concurrently, the environment in which humans exist is undergoing unprecedented transformations. Characterized by climate change and air pollution, this environmental crisis has been identified as one of the most significant public health threats of the twenty first century [3,4]. Nevertheless, these two major trends are not isolated phenomena; they are interconnected through intricate and multifaceted pathways. Key environmental meteorological factors primarily encompass ambient temperature and air pollutants. However, the health implications of temperature exposure cannot be generalized across the globe, as climatic baselines differ substantially by region. Accordingly, temperature exposure is better understood as a relative concept defined by local climate norms rather than as an absolute value [5]. For example, in a nationwide birth cohort study across eight provinces in China, heat events were defined using a range of temperature cutoffs from the 50th to the 97.5th percentile, and the final threshold was set at a daily maximum temperature at or above the 90th percentile of the local temperature distribution or 30 °C for at least one day [6]. Local heat adaptation, urban infrastructure such as air-conditioning availability, and outdoor behavioral patterns may further shape actual exposure and biological response. Outdoor air pollution is a complex mixture, containing particulate matter(PM) and gaseous components such as nitrogen dioxide (NO_2_) and ozone (O_3_). Beyond their inherent physiological toxicity, these components, especially PM, can serve as carriers for heavy metals, persistent organic pollutants, and endocrine disruptors such as phthalates, and these substances carried by PM also become toxic sources of air pollution [7]. Indoors, cigarette smoke and cooking fumes are major components of air pollution. Environmental meteorological factors are increasingly recognized as a pervasive yet insidious exogenous stressor, exerting adverse effects on female reproductive function [8].

Initially, research primarily focused on the direct detrimental effects of heat stress and atmospheric pollutants on pregnancy outcomes. Extensive epidemiological investigations and meta-analyses have substantiated that prenatal exposure to heat constitutes a significant risk factor for various pregnancy-related complications, including miscarriage, preterm birth, stillbirth, and gestational hypertension [9,10]. Similarly, both short-term and long-term maternal exposure to atmospheric pollutants has been significantly associated with early pregnancy loss, reduced live birth rates, and impaired fetal development [11–14]. These findings unequivocally demonstrate that the process of pregnancy maintenance exhibits high susceptibility to heat stress and air pollution.

As research advances, the scientific focus has broadened from isolated pregnancy events to a wider spectrum of reproductive disorders. Emerging evidence suggests potential environmental connections across various conditions, including precocious puberty in adolescents, accelerated decline of ovarian reserve in reproductive-aged women [15,16], and from menstrual cycle irregularities to polycystic ovary syndrome (PCOS) and endometriosis [17–19]. Meanwhile, temperature variation and air pollution can also compromise sperm quality, contributing to male reproductive impairment [20,21].

Successful reproduction relies on the precise coordination of three core elements: competent oocytes, synchronized endocrine signaling, and a receptive endometrium. Accumulating evidence demonstrates that environmental exposures can disrupt each of these critical components. Specifically, air pollution may compromise the blood-follicle barrier, directly impairing oocytes and surrounding granulosa cells, ultimately diminishing oocyte quality and accelerating ovarian reserve depletion [22,23]. Furthermore, it can disrupt the synthesis and metabolism of reproductive hormones, leading to hormonal imbalances and undermining endocrine homeostasis, with similar effects also observed under heat stress conditions [24,25]. Additionally, chronic PM exposure may alter the endometrial gene expression profile and immune microenvironment, thereby compromising endometrial receptivity and contributing to implantation failure [26].

Current research has identified several pivotal molecular hubs mediating these effects. Oxidative stress and inflammatory responses represent common pathways through which environmental exposures initiate damage. Both high temperature and pollutants can disrupt redox homeostasis and trigger inflammation [27,28]. Second, epigenetic dysregulation, serves as a dynamic mechanism responsive to environmental cues, reprogramming gene expression patterns [29]. Finally, the disruption of apoptosis-autophagy balance directly executes functional impairment in reproductive cells and tissues. By interfering with these critical cellular fate decisions, heat stress and air pollution ultimately induce follicular atresia, diminish embryonic developmental potential, and compromise endometrial receptivity [30–32]. (Figure 1)

**Figure 1.**
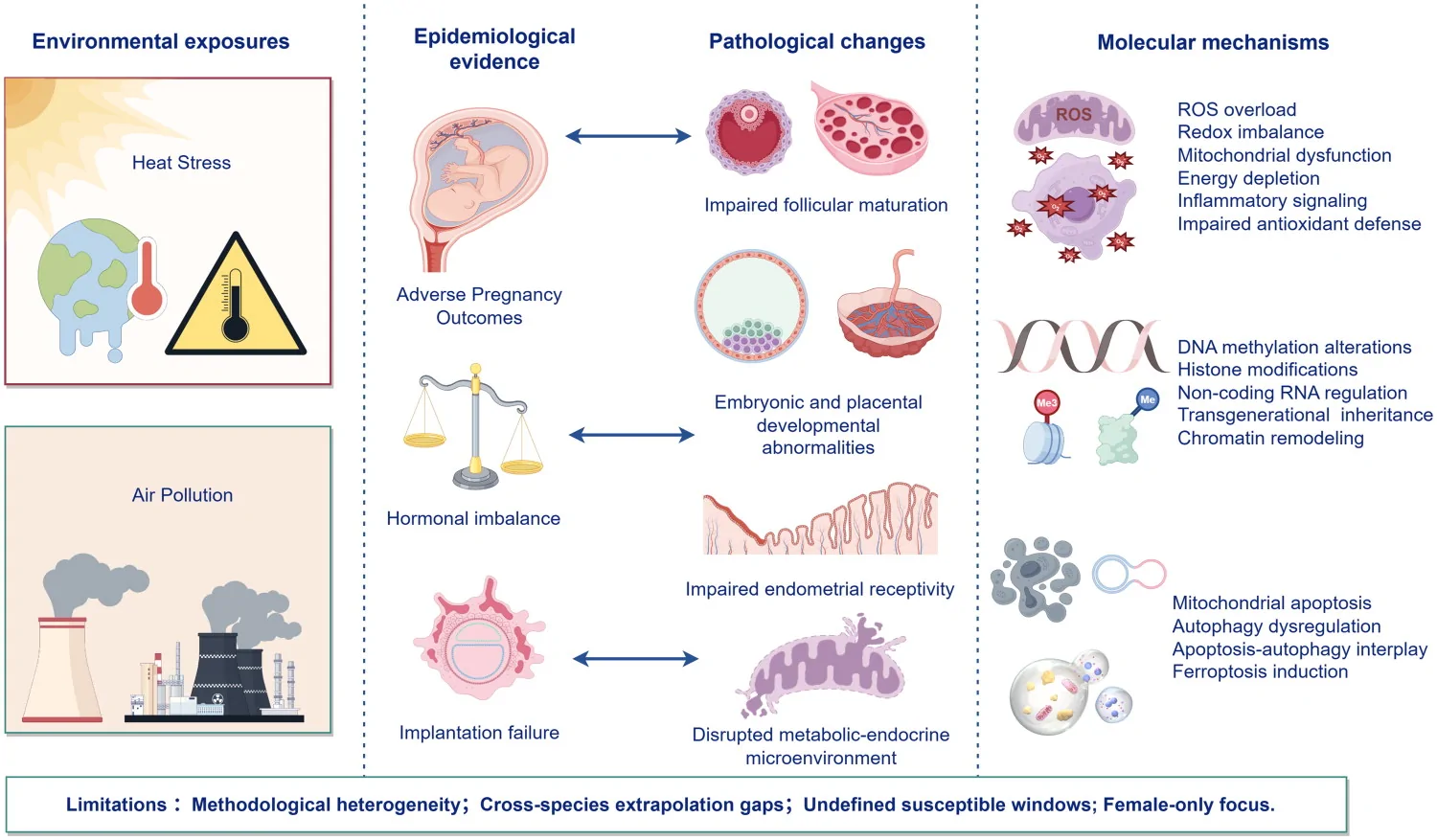
Impacts of Heat Stress and Air Pollution on Female Reproduction.

## 2. Epidemiological evidence linking environmental exposures to female reproductive health

### 2.1. Adverse pregnancy outcomes

The relationship between temperature exposure and adverse pregnancy events generally follows a U-shaped curve. Multiple studies have demonstrated that both extreme heat and cold can reduce early pregnancy viability and live birth rates [33]. Because populations differ in baseline climate and adaptation, many studies use relative rather than absolute temperature [34,35]. A heat event defined by the same relative threshold may therefore correspond to very different absolute temperatures across climatic settings. For example, a locally extreme heat day in Seattle and one in Phoenix may both be rare events for the resident population, yet they differ substantially in absolute temperature [5]. Although this relative approach facilitates cross-regional comparisons, health impacts may still vary by climate. Indeed, a nationwide study of over 5.44 million births in China found that heatwave-associated preterm birth risk differed substantially across climate types [36].

Compared with cold exposure, elevated temperatures appear to have a stronger association with embryo-development-related complications [37,38]. A meta-analysis of 70 studies across 25 countries found that increasing ambient temperatures significantly raise the risks of preterm birth and stillbirth, and that heatwave exposure (defined as two or more consecutive days with temperatures above the 90th percentile) is associated with a 1.16-fold higher risk of preterm birth [39]. Another large-scale population study of 385,337 pregnant women established connections between heat exposure and low birth weight [40]. Additionally, thermal stress has been correlated with reduced crown-rump length in fetuses and adverse cardiovascular development manifested in childhood [41,42]. Notably, the effects of heat exposure on pregnancy outcomes may vary by gestational window. Recent large cohort studies have identified specific susceptible periods, with late pregnancy considered a critical window in several studies, and mid-pregnancy, especially gestational weeks 16–22, also reported as a sensitive period [6,38,43,44]. Terada et al. based on a nationwide birth cohort in Japan, found the highest risk at week 19 [45]. However, the exact susceptible window is still not fully consistent across studies; some suggest a broader window, such as gestational weeks 10–23, whereas others report that the sensitive period differs by pregnancy outcome [44,46]. These discrepancies may reflect differences in exposure definitions, analytic methods, and geographic context. Conversely, extreme cold temperatures also present significant threats to pregnancy outcomes. A comprehensive meta-analysis of 34 studies demonstrated that exposure to temperatures below the 1st percentile of local averages increases risks of adverse delivery outcomes, highlighting the importance of avoiding extreme cold exposure during pregnancy [47]. Moreover, low temperatures show particularly strong associations with maternal pregnancy complications such as gestational hypertension [48].

The reproductive toxicity of atmospheric pollutants has been substantiated by extensive research. Studies demonstrate that short-term exposure to carbon monoxide (CO) and sulfur dioxide (SO_2_) is significantly associated with increased risk of early pregnancy loss, with these effects frequently exhibiting delayed manifestation [49]. In populations undergoing assisted reproductive technology (ART), NO_2_ and PM_10_ exposure adversely impact clinical outcomes by reducing both pregnancy and live birth rates [50]. A large-scale retrospective cohort study further established an inverse correlation between O_3_ exposure and live birth rates [51]. Beyond gaseous components, particulate-phase pollutants—including polycyclic aromatic hydrocarbons from waste incineration and industrial emissions, as well as heavy metals adsorbed onto aerosol particles—constitute additional toxic constituents that compromise pregnancy outcomes [52,53]. Mirroring patterns observed with temperature exposure, the detrimental effects of air pollutants extend beyond primary pregnancy outcomes to encompass serious maternal complications such as gestational hypertension [54], while also exerting adverse impacts on fetal development [55,56].

### 2.2. Reproductive endocrine disorders

The female reproductive endocrine system operates through a sophisticated regulatory network centered on the hypothalamic-pituitary-ovarian (HPO) axis, incorporating complex feedback loops and coordinated by key metabolic factors including thyroid hormones and insulin sensitivity. Environmental exposures are increasingly recognized as disruptive elements to this network, potentially accelerating reproductive aging [17,57,58].

PM plays a pivotal role in endocrine disruption. An epidemiological study in the United States revealed that exposure to PM_2.5_ (PM with aerodynamic diameter ≤ 2.5 μm) and PM_10_ (≤ 10 μm) can interfere with normal pubertal development in girls, leading to alterations in menarche timing [59]. Research conducted in Chinese children further established that long-term PM_2.5_ exposure significantly increases the risk of precocious puberty in girls, demonstrating notable gender specificity [60]. Among reproductive-aged women, PM_2.5_ exposure correlates strongly with reduced oocyte yield [61], suggesting direct adverse effects on ovarian function. A recent meta-analysis of 15,935 pregnant women demonstrated that PM_2.5_ exposure during pregnancy is associated with higher odds of suboptimal vitamin D levels [62]. Vitamin D critically regulates ovarian function, folliculogenesis, and fertility maintenance through multiple pathways, including modulation of AMH production, improvement of insulin sensitivity, and enhancement of endometrial receptivity [63]. Black carbon, a key toxic component of PM_2_._5_, has been detected in ovarian tissue and follicular fluid, posing direct threats to mature oocytes [23]. Additionally, air pollutants demonstrate significant associations with thyroid dysfunction and glucose metabolism disorders [64,65]. As thyroid function and insulin sensitivity serve as crucial regulators of reproductive rhythm and energy homeostasis, their dysregulation amplifies the disruptive impact of pollutants on the reproductive endocrine network. Furthermore, fine particles act as carriers for various synthetic chemicals with endocrine-disrupting properties, which upon inhalation may disrupt maternal-fetal hormonal balance [66].

Other air pollution components have also been linked to endocrine abnormalities. PCOS—characterized by hyperandrogenism, chronic anovulation, and polycystic ovarian morphology—represents a classic reproductive endocrine metabolic disorder. Its core pathological features include insulin resistance and HPO axis dysfunction, significantly compromising fertility and long-term metabolic health [67]. A global study encompassing 171 countries identified air pollutants, particularly NO_2_, as contributors to increased PCOS disease burden [68]. A large longitudinal cohort study in Taiwan, China, which followed 91,803 reproductive-aged women for up to 13 years, further indicated that long-term exposure to SO_2_, NO, NO_2_, NOx, and PM_2.5_ was associated with an increased incidence of PCOS [69].

Concurrently, temperature stress resulting from global warming presents growing concerns. Research indicates that prenatal heat exposure can alter maternal progesterone levels and affect offspring neurobehavioral development, providing novel evidence for early-life programming theories [24]. Multiple studies have shown that gestational glucose metabolism abnormalities are associated with elevated ambient temperature [9]. Among these, the cumulative rise in environmental temperature during several weeks preceding the glucose tolerance test, rather than the mean daily temperature, was a stronger predictor of declining β-cell function and elevated blood glucose [70]. Given insulin sensitivity’s crucial role in maintaining normal ovulation and sex hormone balance, heat exposure indirectly disrupts metabolic-endocrine homeostasis. In addition, studies demonstrate that high temperature exposure may accelerate ovarian reserve depletion, leading to reduced oocyte quantity and quality [71,72].

Collectively, these epidemiological observations provide consistent evidence that ambient high temperature and air pollution are associated with a broad spectrum of adverse reproductive outcomes. However, it is critical to recognize that observational studies are inherently limited by residual confounding, exposure misclassification, and selection bias. Evaluating whether these associations are supported by biological plausibility through pathological and mechanistic evidence is therefore of critical importance.

## 3. Pathophysiology of environmental effects on female reproduction

### 3.1. Follicular, embryonic and placental impacts

Environmental factors can induce impaired follicular maturation and corresponding ovarian structural alterations. Follicular quantity and quality are key indicators of ovarian reserve and function. Multiple studies demonstrate that air pollution significantly disrupts follicular development from primordial to small antral stages, reducing antral follicle count [61,73,74]. A large prospective cohort study established significant correlations between PM_2.5_ exposure and decreased numbers of metaphase II oocytes, two-pronuclear zygotes, and high-quality cleavage-stage embryos, indicating compromised oocyte maturation [75]. Anti-Müllerian hormone (AMH), produced by immature small follicles, plays crucial roles in follicular recruitment and development, serving alongside antral follicle count as a key ovarian reserve marker. Multiple studies have linked air pollution to reduced serum AMH levels, reflecting abnormal folliculogenesis under pollutant exposure [76–78]. In ART populations, air pollution correlates with poor ovarian response and decreased numbers of transferable embryos [79]. Two separate studies revealed that PM_2.5_ exposure induced significant vacuolization, granulosa cell atrophy, and increased follicular atresia in murine ovaries [80,81]. The observed polycystic-like ovarian changes in pollutant-exposed animals corroborate epidemiological links between environmental exposures and PCOS risk [82].

Current animal evidence demonstrates that air pollutants interfere with embryonic developmental potential. A recent study integrating a real-world cohort of 885 first-cycle IVF patients with a litter-controlled mouse experiment revealed that long-term ambient PM_2.5_ exposure significantly impairs blastocyst development. Blastocysts derived from exposed females exhibited a pronounced glycolytic shift with upregulated expression of glycolytic enzymes, accompanied by activation of hypoxia-inducible factor 1-alpha, elevated oxidative stress, suppressed mitochondrial function and tricarboxylic acid cycle activity, and disrupted proteostasis. These findings suggest that PM_2.5_ exposure compromises early embryonic development through the shared mechanisms of metabolic reprogramming, mitochondrial impairment, and proteotoxic stress [83]. Consistent with this, PM_2.5_ exposure *via* intratracheal instillation in a mouse model dose-dependently reduced blastocyst formation and increased embryo degeneration. The resulting blastocysts also showed a lower proportion of OCT4-positive cells and a higher proportion of TUNEL-positive cells, pointing to impaired inner cell mass integrity and elevated apoptosis [84].

The placenta serves as a critical interface between maternal and fetal circulation, playing vital roles in fetal development. Air pollution can rapidly induce pathological changes at multiple levels in the placenta and promote a pro-inflammatory microenvironment [85,86]. Experimental evidence indicates that PM_2.5_ can traverse the placental barrier and that PM_2.5_ exposure is associated with mitochondrial swelling and collagen disruption; PM_2.5_ has also been reported to polarize immune cells toward pro-inflammatory phenotypes and to suppress anti-inflammatory responses. Among air pollutants, NO_2_ shows a strong association with placental vasculopathy [85]. First-trimester exposure correlates with reduced spiral artery area and increased vascular tortuosity, impairing placental perfusion capacity. Although late gestation exhibits compensatory increases in arterial area and arc length, this coincides with reduced branching complexity, indicating compromised vascular remodeling. This combination of early impairment and maladaptive late-stage remodeling likely compromises placental exchange efficiency, ultimately contributing to adverse pregnancy outcomes [85]. Fournier et al. reported that maternal pulmonary exposure to air pollution during late gestation resulted in particle translocation to both placental and fetal tissues, accompanied by reductions in fetal and placental weight [87]. D’Errico et al. further showed that repeated inhalation of titanium dioxide nanoparticles during pregnancy led to titanium accumulation in both the decidual/junctional zone and the labyrinth zone of the placenta, and transmission electron microscopy directly identified particles within syncytiotrophoblast cells [88]. Collectively, these findings support the concept that inhaled nanoparticles can cross biological barriers, accumulate in placental tissue, and potentially disrupt placental function.

Temperature-induced pathophysiological changes primarily manifest as heat stress impacts. Elevated temperatures reduce oocyte quality, with a study of 631 infertile women aged 18–45 years showing each 1 °C increase in 90-day mean maximum ambient temperature before ovarian reserve testing corresponded to a 1.6% decrease in antral follicle count [71]. Consistently, Bonell et al. in a cohort of pregnant subsistence farmers in The Gambia, reported that higher environmental heat stress was associated with signs of fetal strain, including alterations in fetal heart rate and umbilical artery resistance index [89]. Animal studies confirm parallel effects, demonstrating reduced follicular volume, arrested development, and decreased dominant follicle proportions [90,91]. Heat-exposed mice exhibit oocyte-granulosa cell dissociation and disorganized granulosa layers with pyknotic nuclei [91]. Studies in bovine models have helped clarify how heat stress affects reproduction. In females, heat stress can disrupt hypothalamic–pituitary–ovarian signaling, reduce follicular dominance and growth, and impair ovarian steroidogenesis [20,92,93]. At the cellular level, heat stress can induce apoptosis and mitochondrial dysfunction in the follicle [93]. In bovine granulosa cells, heat exposure increases HSP70, HSP90, and BAX expression and activates mitochondrial apoptotic pathways [93]. It also alters the follicular fluid proteome and shifts steroidogenic activity toward progesterone secretion, suggesting abnormal luteinization [94,95]. Together, these findings suggest that the adverse effects of heat exposure during pregnancy extend beyond maternal systemic burden and may also involve impaired uteroplacental perfusion and disrupted placental development, thereby compromising fetal well-being.

### 3.2. Metabolic-endocrine homeostasis disruption

Environmental pollutants induce measurable abnormalities in multiple reproduction-related metabolites. A metabolomics investigation evaluating air pollution’s impact on oocyte maturation identified NO_2_ as exhibiting particularly prominent metabolic disruption, sharing the greatest number of metabolic pathways with oocyte developmental processes [96]. These overlapping biological pathways—including vitamin D3 metabolism, bile acid biosynthesis, and steroid hormone metabolism—are similarly vulnerable to temperature variations, as demonstrated in a bovine *in vitro* cell study [97]. Under air pollution exposure, abnormal levels of pregnenolone and 7-dehydrocholesterol, key intermediates in steroid hormone biosynthesis, demonstrate specific disruption of steroidogenic pathways during oocyte development, with similar findings reported in mouse model [81]. During controlled ovarian stimulation in ART cycles, air pollution correlates with significant alterations in follicular fluid metabolite profiles, particularly affecting biomarkers of oxidative stress and nutrient metabolism, thereby revealing pollutant-induced remodeling of the ovarian microenvironment at the metabolic level [11]. Large-scale nontargeted metabolomic analyses of maternal serum samples identify multiple shared metabolic pathways between air pollution exposure and various adverse pregnancy outcomes, with core pathways again involving steroid/vitamin metabolism, oxidative stress, and inflammatory responses [98,99]. A population study on missed abortion provides direct evidence that PM_2.5_ and its key components trigger metabolic microenvironment imbalance in chorionic villi, disrupting nucleotide metabolites (including 2′-deoxyinosine triphosphate) enriched in purine and pyrimidine metabolism pathways. These disruptions showed significant negative correlations with serum levels of pregnancy-associated plasma protein A, vascular endothelial growth factor, and progesterone—all critical biomarkers for embryonic development [100].

Environmental exposures are closely associated with disruptions in reproductive hormone homeostasis. Multiple clinical studies have demonstrated significant correlations between air pollution and altered serum levels of reproductive hormones. Specifically, prenatal exposure to air pollution has been associated with reduced progesterone levels in high-pollution areas; long-term exposure to PM_2.5_ and its components has been linked to decreased estrogen and increased follicle-stimulating hormone (FSH) levels in female adults; and long-term exposure to air pollution has been associated with diminished AMH levels, although findings on AMH remain debated [24,57,76–78,101]. Parallel effects of thermal stress on metabolic profiles have been observed in female rat models, where heat exposure induces decreased estradiol and progesterone concentrations accompanied by disrupted reproductive cyclicity [102]. These critical hormonal fluctuations can lead to ovulatory dysfunction and interfere with signaling along the HPO axis. Experimental studies in mice have demonstrated that air pollution exposure disrupts endogenous sex hormone balance and induces gonadal axis dysfunction [103]. Airborne PM carrying adsorbed organic compounds, including benzene series and polycyclic aromatic hydrocarbons, has been proposed to act as endocrine-disrupting chemicals (EDCs) capable of interfering with hypothalamic-pituitary-gonadal axis regulation [57]. Obesity-related infertility, characterized by androgen excess from insulin resistance and HPO axis suppression due to leptin resistance, shows strong interactions with environmental factors [104]. Long-term PM_2.5_ exposure has been shown to induce insulin resistance and disrupt glucose metabolism in female rat model [105]. Leptin, an adipocyte-derived hormone that communicates energy storage status to the hypothalamus, shows elevated circulating levels in association with PM_2_._5_ exposure according to multiple epidemiological studies [106,107]. This pollutant-induced hyperleptinemia may initiate a vicious cycle promoting further weight gain and metabolic deterioration.

Environmental exposures can also disrupt maternal-fetal energy metabolism. Research confirms that heat stress directly impairs mitochondrial function in maternal decidual cells and fetal amniotic epithelial cells, manifesting as reduced ATP synthesis and dysregulation of key energy metabolism genes [108]. Subsequent targeted metabolomic analyses revealed specific alterations in energy-related pathways, including fluctuations in tricarboxylic acid cycle intermediates, inhibition of fatty acid oxidation, and disruption of nucleotide metabolism. This energy metabolism dysregulation, coupled with temperature-induced inflammatory activation, collectively promotes the occurrence of adverse pregnancy outcomes.

### 3.3. Implantation microenvironment impairment

Successful embryo implantation depends critically on effective blastocyst attachment to the endometrium. Accumulating evidence indicates that various environmental exposures can compromise this process by impairing endometrial receptivity and disrupting embryo-endometrial interactions.

PM_2.5_ represents a significant environmental stressor interfering with implantation. Jung et al. demonstrated that PM_2.5_ exposure alters endometrial gene expression profiles, inducing a pathological state resembling endometriosis in human endometrial cells, with subsequent confirmation of reduced fertility in murine models [26]. Other investigations have revealed the impact of PM_2.5_ on the immune microenvironment. Shuyin Duan et al. observed endometrial thinning in PM_2.5_-exposed mice, with single-cell sequencing revealing significantly decreased natural killer cell proportions and altered cellular heterogeneity in the endometrium, suggesting compromised maternal-fetal immune tolerance [31].

Cigarette smoke, containing numerous toxic compounds, constitutes a major indoor air pollution source. Endometrial receptivity assessment comparing smoking and non-smoking women showed reduced expression of key receptivity markers in smokers, indicating impaired endometrial function [109]. Among airborne and particulate-associated endocrine-disrupting chemicals, bisphenol compounds have been detected in indoor air and dust, and inhalation of dust represents one of several human exposure routes, alongside diet and dermal contact. Experimental evidence indicates that bisphenol A and its analogues can alter estrogen and progesterone receptor expression in human endometrial cells and inhibit spheroid attachment, suggesting a potential adverse effect on endometrial receptivity. However, human evidence directly linking inhaled or particle-bound bisphenol exposure to impaired endometrial receptivity remains limited [110]. Animal studies provide further mechanistic evidence. One murine investigation directly linked air pollution-induced implantation failure to compromised endometrial receptivity, suggesting particulate matter may adversely affect fertility through multiple pathways: impaired uterine morphological differentiation, reduced corpus luteum number, and dysregulation of window-of-implantation protein expression [111]. Temperature variations similarly pose threats. An *in vitro* bovine endometrial cell study demonstrated that heat stress disrupts normal endometrial immune responses, leading to decreased receptivity and fertility [112], while a mouse study indicated that elevated temperatures suppress *Wnt* signaling pathways critical for endometrial receptivity [102].

## 4. Molecular mechanisms underlying environmental effects

### 4.1. Oxidative stress and inflammatory activation

Multiple studies have documented oxidative stress damage in the female reproductive system following environmental exposure, with elevated levels of oxidative damage biomarkers detected in populations under adverse environmental conditions [11,96,99]. Certain air pollutants, particularly PM_2.5_, can penetrate biological barriers and accumulate in reproductive tissues, directly inducing oxidative injury [22]. The major determinants of nanoparticle translocation across biological barriers, including particle size, surface properties, and protein corona formation, are likely to be highly relevant to penetration of the placental barrier [113]. The essence of oxidative stress lies in the disproportion between reactive oxygen species (ROS) generation and the scavenging capacity of endogenous antioxidant defense systems, leading to disrupted redox homeostasis and subsequent damage to crucial macromolecules including lipids, proteins, and DNA. Notably, high temperature and air pollution frequently exhibit overlapping pathways: intensified photochemical reactions during hot weather elevate ambient pollutant concentrations, while elevated temperatures may modify particulate surface properties, enhancing their biological toxicity. Both heat-induced metabolic acceleration and transition metals/polycyclic aromatic hydrocarbons carried on fine particle surfaces contribute to intracellular ROS bursts. Elevated temperatures induce mitochondrial dysfunction, characterized by increased mitochondrial membrane potential, compromised bioenergetic efficiency, and increased ROS production [114]. Concurrently, stress-induced overexpression of heat shock proteins represents an energy-demanding process that may itself generate additional ROS. Meanwhile, PM_2.5_ and other pollutants can directly function as oxidants or be metabolically converted into free radical precursors that inflict cellular damage. This multi-source oxidative assault rapidly depletes key antioxidants such as glutathione and superoxide dismutase, ultimately overwhelming cellular self-protection mechanisms [81,115].

Oxidative stress subsequently triggers downstream metabolic dysregulation. Mitochondria, serving as both primary sources and initial targets of ROS, undergo functional impairment that not only compromises electron transport chain activity but also disrupts organelle ultrastructure, thereby significantly diminishing the developmental competence of oocytes and early embryos [116]. Aberrations in fundamental processes including the tricarboxylic acid cycle and fatty acid β-oxidation lead to sharply reduced ATP synthesis efficiency, resulting in cellular energy deficiency [117]. This metabolic crisis can forces the interruption of critical biosynthetic pathways, while simultaneously inducing spindle assembly abnormalities and cell cycle arrest [118,119]. Concurrently, metabolites essential for maintaining cellular redox homeostasis, including carnitine and nicotinamide in follicular fluid, become depleted under sustained oxidative stress, further weakening antioxidant capacity [96]. This energy and metabolic disruption triggered by oxidative stress represents an important mechanism underlying dysfunction in female germ cells and embryonic development.

High ambient temperatures significantly elevate levels of inflammatory mediators and upregulate pro-inflammatory gene expression profiles [120]. Consistent with these stress responses, experimental evidence from mice has shown that intermittent heat exposure during late gestation reduces the proportion of the placental labyrinth zone and decreases Vegfa expression, supporting a link between heat stress and impaired placental vascular development [121]. ROS function as potent inflammatory signaling molecules that activate intricate intracellular networks, while simultaneously, activated immune cells further generate substantial additional ROS, establishing a self-perpetuating cycle between oxidative stress and inflammation, as demonstrated in the context of uterine–vascular adaptation during pregnancy [122]. The nuclear factor kappa-B (NF-κB) pathway serves as a central inflammatory signaling hub. Environmental pollutants or their induced ROS can relieve inhibitory constraints on NF-κB heterodimers, enabling nuclear translocation and initiation of transcription for numerous pro-inflammatory cytokines and chemokines, including tumor necrosis factor-alpha, interleukin-6 (IL-6), and interleukin-1β [123]. Additional key signaling pathways involved in the oxidative stress response include the NLR family pyrin domain containing 3 inflammasome activation and nuclear factor erythroid 2-related factor 2-mediated antioxidant response, with the latter shown to control oxidative stress and inflammation-related genes in human myometrial cells [124]. Akhigbe et al. further summarized these conserved heat stress response mechanisms across mammalian reproductive systems, highlighting the broad relevance of oxidative, inflammatory, and stress-adaptive pathways in reproductive tissues [20].

### 4.2. Epigenetic dysregulation

Epigenetic modifications represent a crucial mechanism through which environmental factors exert long-term effects on offspring health, encompassing DNA methylation, histone modifications, and non-coding RNA-mediated regulation.

DNA methylation serves as the most extensively studied environmental epigenetic mechanism. The high DNA synthesis rate and active epigenetic remodeling during embryogenesis make the fetal epigenome a critical interface connecting early environmental exposures with adverse birth outcomes and long-term health risks. Studies report distinct DNA methylation patterns in maternal peripheral blood mononuclear cells and infant cord blood mononuclear cells after gestational PM_2.5_ exposure [29]. These maternal–fetal differences support an association between PM_2.5_ exposure and altered epigenetic profiles across the maternal–fetal interface, but they do not identify the underlying route. The observed differences may reflect direct transplacental effects of PM_2.5_ or particle-bound components, indirect placental responses, maternal systemic effects, or shared exposure-related confounding. Integrated single-cell sequencing and DNA methylation analyses in women exposed to air pollution suggest that air pollution exposure is associated with altered methylation of insulin-like growth factor 2 mRNA-binding protein 1 in women with recurrent pregnancy loss [125]. During critical developmental windows, temperature exposure has been linked to persistent and potentially transgenerational programming effects on energy homeostasis, consistent with methylation changes in key metabolic genes [126].

The placenta represents a primary target for environmentally induced epigenetic modifications. Epigenetic regulation is essential for normal placental growth and function, and altered gene expression during critical developmental windows can disrupt key cellular processes, leading to aberrant placental and fetal development. Multiple studies have reported that prenatal air pollution exposure is associated with distinct DNA methylation changes in placental tissue, which in turn have been associated with pregnancy complications such as preeclampsia [127–130]. Specifically, methylation alterations at *APEX1*, *OGG1*,and *ERCC4* loci impair placental DNA repair capacity [131]. Comparative analysis of methylation patterns before and after air pollution provides valuable insights into underlying molecular pathways. Experimental evidence from pregnant mice exposed to air pollution reveals site-specific DNA methylation changes in placental tissue concurrent with significant activation of IL-6 and JAK/STAT inflammatory signaling pathways. Notably, dietary intervention with an omega-3 rich fish oil diet administered from 8 weeks prior to conception through the entire gestational period partially reversed these pollution-induced effects, including partial reversal of air pollution-induced gene expression changes [132]. Beyond inflammation, air pollution exposures trigger epigenetic modifications affecting lipid metabolism, mitochondrial energy production, and immune regulation [133,134]. Collectively, placental DNA methylation studies suggest that oxidative and nitrosative stress may represent central mechanisms underlying air pollution-associated epigenetic effects. Reactive oxygen and nitrogen species directly damage DNA through strand breaks, base modifications, and cross-linking. Such structural damage may impede DNA methyltransferase recognition and binding and has been proposed to reduce genome-wide methylation levels [135]. These findings highlight the complex interplay between different mechanistic pathways in environmental disruption of female reproductive health.

Histone modifications and non-coding RNA regulation represent additional pivotal pathways through which environmental exposures induce epigenetic changes. Histones, as fundamental structural and functional components of eukaryotic chromatin, undergo post-translational modifications that directly govern gene expression states. Beyond the heat stress-induced alterations in multiple histone variant expressions [136,137], emerging evidence indicates that air pollution can trigger endoplasmic reticulum stress through modified histone profiles, significantly impairing trophoblast cell invasion and fusion capabilities [138]. Single-cell epigenomic analyses further identify specific histone modification markers, including H3K9ac and H3K27ac, as significantly correlated with environmental exposures. These modifications facilitate aberrant developmental defects, placental dysfunction, and adverse pregnancy outcomes [139]. Non-coding RNAs constitute a class of functional RNA molecules that, while not encoding proteins, critically regulate gene expression through influencing RNA splicing and other key biological processes. Maternal NO_2_ exposure can alter substantially the expression profile of long non-coding RNAs in fetal tissues. Mechanistically, maternal NO_2_ exposure induces acquired methemoglobinemia and triggers maternal–fetal hypoxia transmission; the resulting hypoxic microenvironment upregulates HIF-1β, which in turn represses the transcription of hypoxia-responsive long non-coding RNAs [140]. Airborne or particulate-bound endocrine-disrupting chemicals, including phthalates and phenols, also have been reported to disrupt placental function and fetal development through modulation of non-coding RNA expression; however, human evidence for an inhaled or particle-bound exposure pathway remains limited [141].

Transgenerational transmission represents a critical characteristic of epigenetic mechanisms in environmental reproductive toxicity. Prenatal exposure to air pollution alters the methylation status of DNA repair genes in placental tissue [131]. Maternal gestational contact with pollutants can influence offspring development by modifying chromatin accessibility and transcription factor motif activity [142,143]. Prenatal exposure to benzo[a]pyrene, a representative carcinogenic constituent of PM, has been linked to transgenerational female reproductive toxicity, consistent with epigenetic reprogramming, and characterized by diminished ovarian follicle reserve, aberrant male-specific gene expression in female germ cells, and altered DNA methylation at repetitive sequences across generations [144]. Environmental exposures also trigger sex-dependent epigenomic remodeling. Research reveals that male and female infants exposed to air pollution exhibit distinct DNA methylation profiles, with critical windows for methylation changes showing significant sexual dimorphism [145].

### 4.3. Apoptosis and autophagy dysregulation

Apoptosis and autophagy play essential roles in maintaining ovarian function, embryonic development, and pregnancy maintenance. Environmental stressors disrupt this delicate balance, leading to female reproductive dysfunction. Both thermal stress and air pollution can trigger mitochondrial pathway-mediated programmed cell death in female germ cells [84,93,146,147]. The specific mechanisms involve disrupted balance of Bcl-2 family proteins, with pro-apoptotic proteins Bax and Bak undergoing conformational changes and altered expression levels. These proteins form pores in the mitochondrial outer membrane, facilitating cytochrome c release into the cytoplasm. The released cytochrome c then complexes with apoptotic protease-activating factor 1 and caspase-9 to form the apoptosome, which subsequently activates downstream caspase-3, ultimately resulting in DNA fragmentation and cellular demise. Air pollution exposure also induces apoptosis in trophoblasts through alternative pathways. Sulfur dioxide and its derivatives suppress the reactive oxygen species/interleukin-6/signal transducer and activator of transcription 3 signaling axis, leading to cell cycle arrest and apoptosis induction [148]. In uterine tissue, activation of apoptotic pathways may contribute to endometriosis pathogenesis, significantly compromising embryo implantation [26].

Dysfunctional autophagy represents another crucial mechanism through which environmental exposures impair reproductive function. In the ovary, heat stress directly upregulates LC3B-II and ATG12–ATG5 conjugation levels and downregulates p62 expression in granulosa cells, and these changes are clearly correlated with increased follicular atresia and reduced oocyte maturation rates in pigs and mice [149–152]. In the goat endometrium, heat stress has been associated with reduced expression of integrin αvβ3, a receptivity marker, possibly *via* altered autophagic flux [153]. Air pollution disrupts autophagic regulation through oxidative stress pathways, as ROS generated by PM_2.5_ can directly oxidize and modify core autophagy proteins, leading to autophagic interruption. In placental trophoblast cells, impaired autophagy has been associated with delayed clearance of damaged mitochondria and misfolded proteins, which may exacerbate cellular dysfunction and affect fetal growth [84]. However, the involvement of inflammatory pathways such as NF-κB and NLRP3 in this process remains based on inferences from *in vitro* experiments, lacking direct causal validation within placental tissues [152].

The interplay between apoptosis and autophagy holds significant implications for environmental reproductive toxicity. At the molecular level, these two processes interact through multiple key nodes. Beclin-1, a critical autophagy initiation regulator, contains a BH3 domain that enables interaction with Bcl-2/Bcl-xL, and this interaction is modulated by JNK-mediated Bcl-2 phosphorylation [154]. In ovarian granulosa cells, JNK activation promotes the dissociation of the Bcl-2/Beclin-1 complex, thereby releasing Beclin-1 to initiate autophagy [154,155]. Environmental exposures that activate JNK signaling may similarly disrupt this balance, coordinating the regulation of both apoptosis and autophagy. In a porcine model, cyclical heat stress increased ovarian Beclin 1 and LC3B protein levels, indicating autophagy induction in ovarian follicles, while also upregulating BCL2L1 in oocytes and granulosa cells, suggesting a parallel upregulation of anti-apoptotic signals [150]. Furthermore, caspase family proteases can interact with autophagy-related proteins including Beclin-1, thereby coordinating the regulation of apoptosis and autophagy in reproductive cells [156].

Tissue-specific responses represent a hallmark of environmental exposure effects. In ovarian tissue, granulosa cells demonstrate particular susceptibility to heat stress-induced apoptosis, involving disrupted FSH/cyclic adenosine monophosphate signaling and impaired estrogen synthesis [146,157,158]. Granulosa cell apoptosis elevates local levels of oxidants and pro-apoptotic factors, subsequently triggering oocyte apoptosis. Within placental tissue, the specific death modalities adopted by cytotrophoblasts and syncytiotrophoblasts under environmental stress are likely to influence placental functional integrity and fetal development [159].

Emerging evidence reveals that novel programmed cell death pathways further diversify the mechanistic landscape of environmental reproductive toxicity [160]. Environmental stressors can induce ferroptosis, characterized by accumulated lipid peroxides and inhibited glutathione peroxidase 4 activity. In ovarian granulosa cells, ferroptosis disrupts antioxidant defense systems, exacerbating oxidative damage [161]. Importantly, PM_2.5_ exposure and heat stress have been reported to be associated with ferroptosis in ovarian granulosa cells, suggesting that ferroptosis may represent one pathway through which air pollution and thermal stress contribute to ovarian dysfunction [115,162].

## 5. Discussion

This review synthesizes epidemiological, pathological, and mechanistic evidence on how ambient temperature and air pollution affect female reproductive health. The evidence is consistent: heat stress and air pollution are associated with lower ovarian reserve, reduced implantation and live birth rates, higher pregnancy loss, and increased risks of preterm birth and hypertensive disorders. These associations are consistent with pathological findings of impaired follicular development, defective placental vascular remodeling, and reduced endometrial receptivity; mechanistic studies further suggest that oxidative stress, epigenetic reprogramming, and dysregulation of apoptosis and autophagy may contribute to these effects. Nevertheless, several issues limit the interpretability of current findings. One is the lack of standardization in exposure assessment. In epidemiological studies, temperature is often simplified to a single indicator, such as mean temperature, maximum temperature, percentile-based extremes, or cumulative heatwave days. These indicators carry distinct meanings; for instance, heatwaves defined by duration reflect different biological stress than single-day extreme values, and relative thresholds based on local climatology correspond to different absolute temperatures across regions. Yet the rationale for selecting a particular indicator is rarely justified. Air pollution poses even greater complexity, as different pollutants differ in sources, physicochemical properties, and spatiotemporal distributions. However, many studies treat individual components as independent exposures, overlooking their strong collinearity. This practice may misattribute or overestimate the effect of a specific pollutant.

In reproductive medicine, experiments involving pregnant women and fetuses face stringent ethical constraints, making direct validation of molecular mechanisms in human tissues objectively difficult. Consequently, animal models and cell experiments remain the primary approaches for exploring mechanistic pathways. The value of such studies lies first in providing biological plausibility for epidemiological associations. The field has accumulated extensive pathway evidence, covering NF-κB, NLRP3, Nrf2, AMPK/mTOR, and the Bcl-2 family. Notably, these pathways differ in the strength of support. Some have been directly validated in human reproductive tissues. However, others remain primarily only based on animal or cellular observations. Given that species may respond differently to the same exposure, it is essential to distinguish findings that have been corroborated in human tissues from those that are still limited to animal or *in vitro* models. As more physiologically relevant experimental models mature, such as organoids and placental perfusion, they are expected to fill current research gaps.

Population selection is another concern. A considerable proportion of the studies included in this review focus on women undergoing ART. ART offers precise exposure windows, standardized clinical data, and well-defined outcomes, which significantly enhance rigor. However, ART patients do not represent the general reproductive-aged female population. Infertile women may differ in ovarian reserve, endocrine profiles, and inherent susceptibility to environmental insults, limiting the generalizability of conclusions drawn from this subgroup.

Equally noteworthy is that most studies on female reproductive health largely neglect the potential confounding role of the male partner in fertilization, implantation, and subsequent development. Reproduction is a joint outcome of both partners. Sperm quality is also affected by temperature and air pollution, yet couple level interactions such as combined exposure effects or male mediated epigenetic transmission are rarely considered in studies framed around female reproductive health. Restricting covariates to female factors not only risks overestimating the attributable risk of female exposures but also misses critical clues regarding shared pathogenic mechanisms between the sexes, thereby undermining the effectiveness of clinical prevention strategies and ART interventions. Future research urgently needs to incorporate male exposure profiles and partner interaction effects to better approximate real world exposure scenarios.

Within the core evidence, both consistencies and contradictions are observed. The most consistent pattern concerns temperature and pregnancy outcomes: regardless of the temperature metric used, high temperature exposure is positively associated with preterm birth, stillbirth, and low birth weight, a finding replicated across different climatic zones and countries. For air pollutants, the association between PM_2.5_ and diminished reproduction reserve is relatively robust. However, heterogeneity is equally prominent. Some associations are significant only in specific populations or detectable only within particular exposure windows, and findings for the same exposure-outcome pair are not always consistent. Such inconsistencies may stem from differences in exposure assessment methods, with spatial resolutions ranging from 10 km to 250 m and exposure windows varying from single-day lags to multi-year cumulative averages, or from differences in baseline population characteristics, such as age composition, infertility etiology, and socioeconomic status.

Several common methodological limitations also exist in current studies. First, most epidemiological studies rely on residential address-based exposure estimates without incorporating individual activity patterns or indoor/outdoor time allocation, leading to nearly ubiquitous exposure misclassification. Second, exposure concentrations and routes in animal or cellular mechanistic studies may not reflect real-world conditions, and direct human tissue validation is scarce. Third, inadequate control for co-exposures and confounders, such as simultaneous adjustment for temperature, humidity, and multiple pollutants, may bias independent effect estimates. Fourth, identification of susceptible windows varies considerably across studies because of differences in exposure definitions, temperature thresholds, statistical modeling strategies, and population heat adaptation levels. This variation warrants caution when interpreting critical windows across studies and underscores the need for future research to refine window investigation under a unified analytical framework.

Traditionally, large-sample randomized controlled trials are positioned at the apex of the evidence hierarchy. However, this framework may be less applicable to reproductive health research, because interventions involving pregnant and periconceptional women are often ethically untenable. A more appropriate criterion is whether existing evidence converges across multiple dimensions: consistency across countries, a dose–response gradient, and biological plausibility from molecular mechanisms. By these criteria, the evidence linking temperature and air pollution to adverse female reproductive outcomes is consistent, with concordant trends observed across diverse populations, exposure metrics, outcomes, and experimental systems. This convergence strengthens the biological plausibility of a causal link, yet it should be recognized that convergence itself does not constitute causal proof. Given the limitations discussed above, we propose three priority directions for future research. First, exposure assessment standards should be harmonized and refined, including the development of reporting guidelines for pollutant and temperature metrics, improved spatiotemporal resolution, and multicenter collaborations to enhance comparability. Future studies should preselect candidate windows based on biological plausibility and conduct multicenter joint analyses under standardized protocols to distinguish genuine biological susceptibility from methodological artifacts. Second, more physiologically relevant *in vitro* models, such as organoids, should be employed to explore molecular mechanisms, thereby circumventing ethical barriers to direct interventions in pregnant and periconceptional women and enhancing the translational relevance of experimental findings. Third, incorporating long-term, large-sample healthy populations and couple-level exposure assessments will substantially strengthen the robustness of conclusions and facilitate their generalizability to the general population.

## 6. Methods

This article is a narrative review; the literature search was intended to support a structured narrative synthesis. We searched three major databases: PubMed, Web of Science and Embase. For epidemiological studies, the search period was limited to the past five years (January 2021 to July 2026), whereas mechanistic studies, including animal experiments and cell/molecular studies, were searched over the past ten years from 2016 onward, given the relatively smaller volume of such literature and the need to ensure adequate coverage of core pathway evidence. Search terms covered both environmental exposure categories, including air pollution, particulate matter, PM_2.5_, ozone, nitrogen dioxide, heat stress, ambient temperature, climate change, and endocrine-disrupting chemicals, and female reproductive health outcome categories, including fecundity, ovarian reserve, ovarian function, oocyte quality, granulosa cells, pregnancy outcomes, preterm birth, low birth weight, stillbirth, preeclampsia, gestational diabetes mellitus, polycystic ovary syndrome, endometriosis, menstrual cycle, placental methylation, and implantation receptivity, supplemented by Boolean operators and MeSH terms for expanded retrieval, and complemented by manual back-tracking of references from included articles. Key references recommended by the reviewer and classic mechanistic studies identified through back-tracking that fell outside the above time frames were also individually included. The inclusion criteria were: (1) original studies in humans or mammals, including cohort, case-control, cross-sectional, randomized controlled trials, animal experiments, and *in vitro* studies, or systematic reviews and meta-analyses; (2) investigations of associations between environmental exposures and female reproductive health outcomes; (3) provision of quantitative or qualitative evidence on exposure-outcome relationships or biological mechanisms. The exclusion criteria were: (1) non-mammalian studies; (2) conference abstracts, editorials, case reports, and other non-original articles; (3) non-English publications; (4) studies not reporting female reproductive health outcomes; (5) studies not involving the environmental exposures of interest in this review. All articles were independently screened by all authors, with disagreements resolved through discussion; data on study design, population, exposure assessment, outcome measures, main findings, and mechanisms were then extracted from eligible studies. Given the substantial heterogeneity in study design, exposure assessment, and outcome measurement across the included studies, we did not conduct formal quality assessment or meta-analysis, but instead organized the evidence thematically.

Consistent with this narrative approach, and given the varying levels of evidence across the primary original studies, the strengths and limitations of each study should be taken into consideration. A summary of the core evidence in this review is provided in Supplementary Table 1.

## 7. Conclusion

High temperature and air pollution are associated with adverse effects on female reproductive health through interconnected epidemiological, pathological, and molecular pathways. Epidemiological evidence consistently associates these exposures to clinical reproductive impairments, supported by pathological findings of disrupted follicular development, placental vascular remodeling, and endometrial receptivity. These effects may be mediated by oxidative stress, epigenetic dysregulation, and disrupted apoptosis and autophagy; mechanistic studies implicate these pathways, although human causal evidence remains limited. Future research should prioritize standardized exposure assessment, long-term prospective cohorts to identify vulnerable populations and critical windows, and translational validation of preclinical findings to inform public health policies and clinical strategies.

## Supplementary Material

Supplemental Material

## Data Availability

There is no data associated with this research.
